# Treatment of pulmonary hypertension

**DOI:** 10.1186/1824-7288-40-S2-A51

**Published:** 2014-10-09

**Authors:** Paolo Tagliabue, Tiziana Fedeli

**Affiliations:** 1 Neonatology and Neonatal Intensive Care, MBBM Foundation, Monza, Italy

## 

The therapeutic approach to the management of pulmonary hypertension (PH) is based on strategies to decrease pulmonary vascular resistance (PVR) whilst ensuring optimal cardiorespiratory support to improve oxygenation. The goal is to maintain appropriate systemic blood pressure, ensure oxygen release to tissues and minimize lesions induced by oxygen and ventilation.

Inhaled nitric oxide (iNO) remains the mainstay of treatment for this condition. As an inhaled agent it reaches the alveolar space and diffuses into the vascular smooth muscle of the adjacent pulmonary arteries where it causes vasodilation by increasing guanosine monophosphate (cGMP) levels without affecting systemic vascular tone. Although iNO significantly reduces the need for extracorporeal membrane oxygenation, almost 25-40% of iNO-treated infants are considered iNO non–responders.

A complementary vasodilatory pathway in the lung is mediated by cyclic adenosine monophosphate (cAMP); prostacyclin stimulates adenylyl cyclase in vascular smooth muscle cells and causes an increase in intracellular cAMP and vasodilation of the systemic and pulmonary circulatory systems. If given as an inhaled drug the vasodilatory effects of prostacyclin tend to be limited to the pulmonary circulation, making this strategy appealing when acute pulmonary vasodilation is needed. [1]

Inhibition of the cGMP-degrading phosphodiesterase (PDE5) and inhibition of the cAMP-degrading phosphodiesterase (PDE3) are two other promising therapies. Sildenafil is a PDE5 inhibitor, the predominant PDE isoform in the lung responsible for the breakdown of cGMP. It acts by enhancing NO-mediated vasodilation and may facilitate iNO discontinuation in infants with critical illness.[2] Milrinone is a PDE3 inhibitor with inotropic and vasodilatory effects; it improves the left ventricular cardiac function both directly and by reducing systemic afterload and exerts also important effects on the pulmonary vasculature by reducing PVR. It may be a plausible agent for treating patients with PH and impaired myocardial function. [3]

One of the most potent vasoconstrictors described in the pulmonary vasculature is Endothelin-1 (ET-1). Inhibition of ET-1 mediated vasoconstriction could be achieved by administration of an endothelin receptor antagonist (Bosentan). Bosentan lowers pulmonary artery pressure and PVR in children with diverse causes of PH and may improve oxygenation in neonates with persistent pulmonary hypertension; it has also been successfully used as an adjunctive treatment for children receiving long-term prostacyclin therapy.

## Conclusion

Although a great deal of progress has been made in recent decades in PH treatment, it remains a devastating illness that requires further studies to adapt the therapy to pediatric lung and its peculiar vasculature.
